# Distinct roles for lateral and medial rostral prefrontal cortex in source monitoring of perceived and imagined events

**DOI:** 10.1016/j.neuropsychologia.2007.12.029

**Published:** 2008

**Authors:** Martha S. Turner, Jon S. Simons, Sam J. Gilbert, Chris D. Frith, Paul W. Burgess

**Affiliations:** a Institute of Cognitive Neuroscience and Department of Psychology, University College London, 17 Queen Square, London WC1N 3AR, UK; b Department of Experimental Psychology, University of Cambridge, Downing Street, Cambridge CB2 3EB, UK; c Wellcome Trust Centre for Neuroimaging, Institute of Neurology, University College London, 12 Queen Square, London WC1N 3BG, UK

**Keywords:** Functional magnetic resonance imaging (fMRI), Cognitive control, Source memory, Reality monitoring, Self-referential processing, Confabulation

## Abstract

Rostral prefrontal cortex (PFC) is known to be involved in source memory, the ability to recollect contextual information about an event. However it is unclear whether subregions of rostral PFC may be differentially engaged during the recollection of different kinds of source detail. We used event related functional MRI to contrast two forms of source recollection: (1) recollection of whether stimuli had previously been perceived or imagined, and (2) recollection of which of two temporally distinct lists those stimuli had been presented in. Lateral regions of rostral PFC were activated in both tasks. However medial regions of rostral PFC were activated only when participants were required to recollect source information for self-generated, “imagined” stimuli, indicating a specific role in self-referential processing. In addition, reduced activity in a region of medial ventro-caudal PFC/basal forebrain was associated with making “imagined-to-perceived” confabulation errors. These results suggest that whilst the processing resources supported by some regions of lateral rostral PFC play a general role in source recollection, those supported by medial rostral PFC structures may be more specialised in their contributions.

## 1 Introduction

Memory for an event involves not only the ability to remember the event itself, but also the ability to recollect detailed information about when, where and how that event was encountered. This ability to attribute mnemonic representations to particular sources on the basis of their qualitative characteristics has been termed “source monitoring” (e.g. Johnson, Hashtroudi, & Lindsay, 1993). Functional imaging studies of source monitoring reliably activate regions of the rostral prefrontal cortex (PFC), principally Brodmann area 10, indicating a role for this region in the recollection of source details (Cansino, Maquet, Dolan, & Rugg, 2002; Dobbins, Foley, Schacter, & Wagner, 2002; Fan, Snodgrass, & Bilder, 2003; Mitchell, Johnson, Raye, & Greene, 2004; Nolde, Johnson, & D’Esposito, 1998; Ranganath, Johnson, & D’Esposito, 2000; Raye, Johnson, Mitchell, Nolde, & D’Esposito, 2000; Rugg, Fletcher, Chua, & Dolan, 1999). However, little is known about whether rostral PFC (also often termed anterior PFC or frontopolar cortex in the memory literature) is involved in recollection of source in general, or whether subregions of rostral PFC may be differentially engaged by the recollection of different kinds of source detail.

This uncertainty over the role of rostral PFC may be because the precise regions of rostral PFC that are activated across studies of source memory tend to vary. One possible explanation for these findings is that the differences in activation reflect the use of different source memory paradigms. The term “source monitoring” has variously been used to describe the recollection of several different types of contextual detail, for example *where* an item was encountered (spatial context) or *when* an item was encountered (temporal context). But it has also been used to refer to recollection of the type of cognitive processing that occurred when an item was previously encountered, for example recollection of which of two tasks (e.g. counting letters in a target word versus making a pleasantness rating) was previously undertaken with a stimulus at study (task context). Studies by Simons and colleagues have indicated that regions of rostral PFC may be differentially activated by these different types of source monitoring. In two studies, participants viewed famous faces or words, and were asked to make either semantic or pleasantness judgements about them. Recollection of which judgement had been made (task context) was compared to recollection of which side of the screen the stimuli had been presented (spatial context; Simons, Owen, Fletcher, & Burgess, 2005) and to recollection of which of two temporally distinct lists the stimuli had been presented in (temporal context; Simons, Gilbert, Owen, Fletcher, & Burgess, 2005). Regions of lateral rostral PFC were activated in recollection of task, spatial and temporal context, indicating a non-specific role in context recollection. However medial regions of rostral PFC were more sensitive to retrieval of task context than spatial or temporal context. Simons and colleagues inferred that these medial regions were specifically involved in the retrieval of self-generated information, i.e. the internal nature of representations involved in task performance, rather than external spatial or temporal context (see also Dobbins & Wagner, 2005).

If some subregions of medial rostral PFC are particularly sensitive to the retrieval of self-generated information, reality monitoring paradigms may be a particularly useful tool with which to explore specialisation of source memory functions within rostral PFC. This is because reality monitoring involves distinguishing self-generated thoughts, such as imaginings and dreams, from events that have transpired externally, in the outside world (Johnson et al., 1993; Johnson, 1997). If some subregions of medial rostral PFC are indeed particularly involved in the retrieval of internally generated information, then one might predict greater activation in this region during reality monitoring tasks, compared with other forms of source memory task. In support of this hypothesis, Simons, Davis, Gilbert, Frith, and Burgess (2006) conducted an fMRI study in which participants either viewed or imagined words at study, on either the left or right of a monitor screen. At test, they were asked either to recall whether words had been perceived or imagined during the study phase (reality monitoring task), or to recall which side of the screen words had been presented on (spatial context task). As predicted by the earlier studies, they found that lateral regions of rostral PFC were involved in both source tasks, but that medial rostral PFC was more associated with reality monitoring judgements than recollection of spatial context.

In a related study, Vinogradov et al. (2006) compared recollection of source for words that had been viewed embedded in sentences (externally generated) to source recollection of words that participants had generated themselves in order to complete a sentence (self-generated). At test, subjects were asked to identify whether words had been generated by the examiner, or self-generated by the participant. Rather than compare different source tasks to each other as Simons and colleagues had done, they directly compared activation associated with recollecting the source of self-generated words to that associated with recollecting the source of externally generated words, and found that medial rostral PFC activation was specifically associated with source recollection for self-generated words. Their results raise the intriguing possibility that medial regions of the rostral PFC may be sensitive not only to the type of source task being conducted, but also to the type of information that this task is being conducted upon (internally generated or externally presented).

The aim of the present study was therefore to address two questions. First, we set out to replicate previous results indicating that lateral and medial regions of rostral PFC may be differentially sensitive to recollection of different types of source. Second, we aimed to establish whether medial rostral PFC activation is modulated to a greater extent by the type of source task that is being conducted, or the nature of the memory product it is being conducted upon. As regards the first question, the studies reviewed above suggest that lateral regions of rostral PFC are recruited during recollection of source information irrespective of the type of source monitoring that is required. Medial regions, in contrast, seem to be particularly activated by source tasks that require recollection of self-generated information, for example in reality monitoring tasks. Reality monitoring tasks have so far only been contrasted with recollection of spatial context (Simons et al., 2006), however the same pattern of results should be evident if reality monitoring is contrasted with another type of contextual detail, e.g. *when* a stimulus was presented. The present study therefore compared reality monitoring judgements to recollection of temporal context. We presented perceived and imagined stimuli in two temporally distinct lists, and contrasted recollection of perceived/imagined source to recollection of temporal source. If lateral regions of rostral PFC are involved in recollection of source in general then they should be activated when recollecting whether stimuli were perceived or imagined, and when recollecting which list the stimuli were encountered in. In contrast, we would predict that medial regions of rostral PFC should be activated more during recollection of perceived/imagined status than of list. In reference to the second question, previous results make slightly different predictions about whether medial rostral PFC activation is modulated to a greater degree by the type of source task that is being conducted (i.e. reality monitoring versus temporal context), or the nature of the memory product it is being conducted upon (monitoring of internally generated versus externally presented information). Therefore source monitoring of previously imagined items was directly contrasted to source monitoring of previously perceived items in order to explore the contributions of these two factors to the engagement of medial rostral PFC.

## 2 Methods

### 2.1 Participants

Sixteen right handed native speakers of English (5 male, 11 female), with normal or corrected-to-normal vision, took part in the experiment. The volunteers (mean age = 26.2 years, range 19–36 years) were screened using a comprehensive medical questionnaire and informed consent was obtained prior to taking part. Participants were selected on the basis of achieving at least 70% accuracy during a practice session of two study and test blocks in a prior testing session (four volunteers were excluded for poor performance at this stage).

### 2.2 Design and materials

The stimuli consisted of 128 nouns between 3 and 5 letters long, which were used as target items in the study and test phases. In addition 2 sets of 64 nouns between 3 and 5 letters long were used as new items in the test phase.

**Fig. 1:**
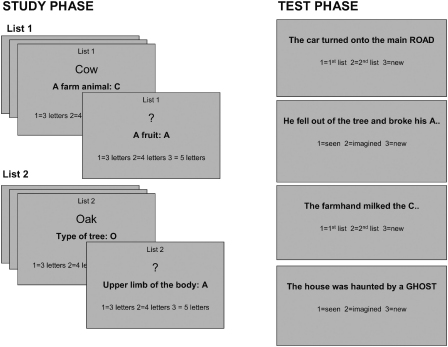
Examples of the stimuli used during study and test phases. In the study phase two temporally distinct lists of items were presented. In each list participants viewed either a clue and target word, or a clue and a question mark, prompting them to imagine the target word. In both cases they were instructed to count the number of letters in the target word. In the subsequent test phase they either viewed or were prompted to imagine target words embedded in sentences, and carried out one of two source tasks: either to recollect whether the word had been presented in the first or second list of the study phase (temporal source task), or whether the word had been seen or imagined in the study phase (perceived/imagined source task).

There were 16 blocks in the experiment, which alternated between study and test blocks (Fig. 1). Each study phase comprised two lists of 8 words each. Within each list half of the words were presented on the screen, while participants were prompted to imagine the other half. Each test phase comprised 32 trials, of which 8 assessed memory for whether the item had previously been perceived or imagined (P/I source), 8 assessed memory for which of the two study lists items had appeared in (temporal source), 8 were “new” trials involving presentation of non-studied items, and 8 were fixation trials. Again half of the target words were presented, and half were imagined by participants. This manipulation was introduced to control for potential priming effects arising when previously perceived items were perceived again at test. Thus there were three experimental factors: study condition (perceive or imagine), test condition (perceive or imagine), and source task (P/I or temporal).

Four different versions of the task were created, which systematically counterbalanced whether items were perceived or imagined at study, whether they were presented in list 1 or 2 at study, whether they were perceived or imagined at test, and which source task was conducted at test (P/I or temporal). New items were drawn from set one for half the participants and from set two for the remaining half. Within each study and test phase, items were pseudo-randomised such that no more than 3 consecutive trials were of the same condition. Words assigned to each condition were matched for Kucera-Francis frequency (Wilson, 1988) and word length.

### 2.3 Procedure

Each study phase consisted of two lists of eight items each, with the beginning of each list signalled by a “List 1” or “List 2” marker. In each study trial, a clue appeared in the centre of the screen along with the first letter of the target word, e.g. “A farm animal: C” Immediately above the clue was either the target word, e.g. “cow” (perceive condition) or a question mark, prompting subjects to imagine the target word (imagine condition). In both conditions participants were instructed to count the number of letters in the target word. A cue at the bottom of the screen (“1 = 3 letters, 2 = 4 letters, 3 = 5 letters”) reminded participants of which response button to press on a button box. They were given 5 s to make their response. A cue at the top of the screen reminded participants which list (list 1 or list 2) was currently being presented.

Test phases followed immediately after each study phase and consisted of all 16 target items from the study phase, along with 8 new items. In each test trial a sentence appeared in the centre of the screen. The sentence either contained the target word in upper case (perceive condition), or contained the first letter of the target word followed a series of dots representing the missing letters (imagine condition). Participants were required to view or imagine the target word and conduct one of two source memory tasks—either determining whether the target item had been perceived or imagined at study (P/I source task), or determining in which of the two lists the target item had been presented at study (temporal source task). A cue at the bottom of the screen indicated which source task was required and which response button to press. The response instructions for P/I source trials read “1 = seen, 2 = imagined, 3 = new”, whilst the response instructions for temporal source trials read “1 = 1st list, 2 = 2nd list, 3 = new”. Participants were given 6 s to make their response.

To increase the efficiency of the event-related fMRI design, the inter-trial interval in both study and test phases was jittered according to an exponential distribution (Henson, 2003) between 480 ms and 1080 ms, and additional fixation periods lasting 6 s were pseudo-randomly interspersed between test trials. Thus although trials were separated by relatively short intervals, as the order of trial types was randomised BOLD response associated with each trial type could be distinguished (Henson, 2003, 2006).

### 2.4 Imaging acquisition and data analysis

A 3T Siemens Allegra system was used to acquire echo-planar functional images (TR = 2.34 s, 64 × 64, 3 mm × 3 mm pixels, TE = 30 ms, 36 sequential axial slices oriented at approximately 30° to the AC-PC transverse plane in order to reduce signal dropout in orbitofrontal lobes, 2 mm thickness, 1 mm inter-slice gap, 4 sessions each of 271 acquisitions). The first 6 volumes from each session were discarded to allow for T1 equilibration.

Data were pre-processed and analysed using SPM2 (Wellcome Department of Imaging Neuroscience, London). Images were first corrected for motion by realigning all images with respect to the first and re-sampling all slices in time to match the middle slice to correct for differences in slice acquisition timing. The realigned images were then normalised to an EPI template in MNI stereotactic space (Cocosco, Kollokian, Kwan, & Evans, 1997). Normalised images were re-sampled into 3-mm cubic voxels and then spatially smoothed with an 8-mm FWHM isotropic Gaussian kernel. A high pass filter of 1/128 Hz was used to remove low-frequency noise, and an AR(1) + white noise model corrected for temporal autocorrelation.

Random effects statistical analysis was undertaken in two stages. In the first stage, event types for each session were modelled by convolving onset times with a canonical haemodynamic response function. The fMRI analysis model included correct trials only. Parameters for each regressor were estimated using a subject-specific model, with movement parameters in the three directions of motion and three degrees of rotation included as confounds, and covariates representing the mean session effects. Within each study session there were 2 regressors (perceived and imagined). Within each test session there were 11 regressors, representing the eight event types of interest (crossing study status, test status and source task), as well as new items (perceived and imagined modelled separately) and fixation trials.

Linear contrasts were used to obtain subject-specific estimates for each of the effects of interest. The following contrasts were specified: For the study phase data the contrast “Perceived versus Imagined” was specified. For the test phase data investigation of the three experimental factors was enabled by specifying the following contrasts: (1) the effect of source task: (a) Any Source Task > New Items; (b) P/I Source > New Items; (c) Temporal Source > New Items; (d) Temporal Source versus P/I Source. (2) The effect of study condition: Any Source Task on Items Perceived at Study versus Any Source Task on Items Imagined at Study. (3) The effect of test condition: Any Source Task on Items Imagined at Test > Any Source Task on Items Perceived at Test. The interaction between study status and source task was also explored with the following contrasts: (a) [Imagined at study (P/I–Temporal Source)] − [Perceived at study (P/I–Temporal Source)]; (b) [Perceived at study (P/I–Temporal Source)] − [Imagined at study (P/I–Temporal Source)]. These estimates were entered into the second stage of analysis treating subjects as a random effect, using one sample *t*-tests across subjects. Statistical parametric maps for each contrast were characterised using an uncorrected height threshold of *p* < 0.001. The peak locations of significant cluster maxima of at least ten contiguous voxels were localised on an averaged structural scan, with approximate Brodmann areas obtained from the Talairach and Tournoux (1988) atlas, after using a non-linear transform of MNI to Talairach coordinates (http://imaging.mrc-cbu.cam.ac.uk/imaging/MniTalairach).

## 3 Results

### 3.1 Behavioural results

Accuracy and reaction time data for the study and test phases are displayed in Table 1. In the study phase there were behavioural differences between the perceive and imagine conditions, with significantly greater accuracy (*t* = 8.9, d.f. = 15, *p* < 0.001) and faster reaction times (*t* = −5.8, d.f. = 15, *p* < 0.001) associated with counting the number of letters in perceived versus imagined words. Turning to the test phase, analysis of accuracy revealed no main effect of whether target words were perceived or imagined at either study or test, but there was a main effect of source task with significantly greater accuracy in the P/I source task than in the temporal source task (*F*_(1,15)_ = 6.72, *p* = 0.02). Analysis of reaction time in the test phase revealed a main effect of source task (slower reaction times associated with the P/I source task *F*_(1,15)_ = 15.41, *p* = 0.001), a main effect of test condition (slower reaction times associated with items that were imagined at test: *F*_(1,15)_ = 16.76, *p* = 0.001), and a test condition × source task interaction with a greater reaction time difference between the P/I and temporal source tasks when items were imagined at test (*F*_(1,15)_ = 10.92, *p* = 0.005). In order to control for differences in performance between the tasks, the fMRI data were examined for all possible influences of difficulty by correlating signal change with both rt and accuracy in any contrasts which involved behavioural differences (see neuroimaging results below).

### 3.2 Neuroimaging results

#### 3.2.1 Study phase

Comparison of the study phase task (counting the number of letters in the target words) for perceived versus imagined words revealed significantly greater activation in several regions including bilateral frontal, bilateral parietal and right occipital cortex. Greater activation for imagined than perceived words was seen in left frontal, left temporal and bilateral occipital regions. A full list of study phase activations is available from the authors on request.

#### 3.2.2 Test phase

More important for the study hypotheses is the data relating to the test phase. The factors of interest were (1) the effect of source task (i.e. comparing recollection of which list the stimuli had been presented in with recollection of whether stimuli had been perceived or imagined at study), and (2) the effect of study condition (i.e. comparing source recollection for items which had been perceived at study with source recollection for items that had been imagined at study). Analysis of test condition (comparing source recollection for items which were currently being perceived to source recollection for items that were currently being imagined) revealed no supra-threshold frontal activations in either main effects or interactions, therefore these conditions are collapsed in the following analyses.

##### 3.2.2.1 Recollection of perceived/imagined and temporal source versus new items

**Fig. 2:**
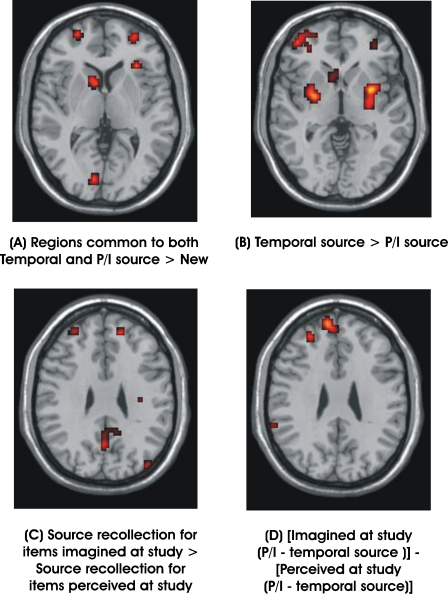
Regions of rostral PFC activated in the critical contrasts. Activations in contiguous regions of bilateral rostral PFC were observed in comparisons of (A) Recollection of Perceived/Imagined and Temporal Source > New Items, and (B) Recollection of Temporal Source > Recollection of Perceived/Imagined Source. These regions are proposed to be involved in several different types of source recollection. By contrast medial regions of rostral PFC were only activated in comparisons of (C) Source Recollection for Items Imagined at Study > Source Recollection for Items Perceived at Study and (D) the interaction term [Imagined at Study (P/I–Temporal source)] − [Perceived at Study (P/I–Temporal source)]. These regions are assumed to be preferentially involved in source recollection involving self-generated information.

In order to find regions involved in correct recollection of both P/I and temporal source information, the two conditions (P/I Source > New and Temporal Source > New) were inclusively masked, both thresholded at *p* = 0.001 uncorrected, and using an extent threshold of 10 voxels. This analysis is equivalent to a conjunction analysis, using the “conjunction null” as recommended by Nichols, Brett, Andersson, Wager, and Poline (2005). Correct recollection of both types of source information provoked activation in a number of areas that closely replicated previous studies of source recollection (Cansino et al., 2002; Dobbins et al., 2002; Dobbins & Wagner, 2005; Simons, Owen, et al., 2005; Simons, Gilbert, et al., 2005; Simons, Henson, Gilbert, & Fletcher, 2008). As can be seen in Table 2, regions activated included bilateral rostrolateral PFC (BA 10), left dorsolateral PFC (BA 9/46), bilateral inferior PFC (BA 47), and bilateral inferior parietal cortex (BA 40). The bilateral rostrolateral PFC activations are shown in Fig. 2A.

##### 3.2.2.2 Recollection of temporal source versus perceived/imagined source

Direct comparison of regions more active in recollection of temporal source than P/I source showed activation in the same regions of bilateral rostrolateral PFC as reported above (see Table 3 and Fig. 2B). This reflected the fact that activation in these regions was greater in amplitude and extent in the temporal source task > new contrast, although there was overlap with the regions activated in the P/I source task > new contrast. As noted in Section 3.1 the temporal source task was associated with lower accuracy and faster reaction times than the P/I source task. However, activation in neither right nor left lateral BA 10 regions was significantly correlated with accuracy or reaction time across participants, indicating that activation in this area did not vary as a function of task difficulty or effort. The reverse contrast, comparison of P/I source judgements to temporal source judgements, revealed no supra-threshold regions of activation associated more with recollection of P/I source information.

##### 3.2.2.3 Recollection of source for items perceived at study versus recollection of source for items imagined at study

We next examined the main effect of study condition, combining across source tasks (P/I and temporal) to explore the regions involved in carrying out any form of source judgement on items imagined at study versus items perceived at study. This revealed several regions particularly involved in making source judgements about items imagined at study (see Fig. 2C and Table 4). These included a region of left lateral rostral PFC (BA 10), which was located rather more dorsally than those activated in the previous contrasts, and a region of right medial rostral PFC (BA 9/10) similar to that previously reported by Vinogradov et al. (2006) as being more associated with reality monitoring of self-generated words. No regions were more active when making source judgements about items perceived at study than items imagined at study.

##### 3.2.2.4 Interaction of source task (perceived/imagined versus temporal) and study condition (perceived versus imagined)

Further interesting results emerged from the interaction of source task and study condition (see Fig. 2D and Table 5). The interaction [Imagined at study (P/I–temporal source)] − [Perceived at study (P/I–temporal source)] revealed activation in the same region of left lateral rostral PFC associated with recollection of source for items imagined at study (Fig. 2C), but also a region of left medial rostral PFC. This is reminiscent of medial BA 10 activations reported previously in association with source tasks tapping retrieval of self-generated rather than externally generated source (Dobbins & Wagner, 2005; Simons, Owen, et al., 2005; Simons, Gilbert, et al., 2005; Simons et al., 2006; Vinogradov et al., 2006). This contrast also activated a region of medial ventro-caudal PFC (BA 25). The reverse interaction term yielded no supra-threshold clusters.

**Fig. 3:**
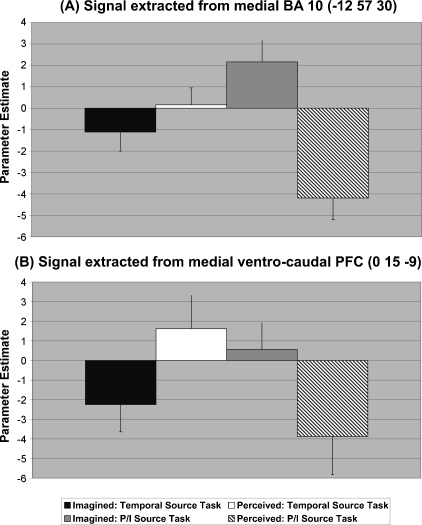
Mean parameter estimates for medial BA 10 and medial ventro-caudal PFC for the temporal and P/I tasks according to study condition (perceived or imagined). Bars represent mean parameter estimates and error bars represent standard error of the mean.

In order to interpret this interaction, parameter estimates for the two medial activations (medial BA 10 and medial ventro-caudal PFC) were extracted from 5 mm-radius spheres centred on the peak coordinates for each condition, and are plotted in Fig. 3. The figures reveal an interesting pattern in that the involvement of these regions in the P/I versus the temporal source task depends upon whether items were perceived or imagined at study. For items imagined at study these regions were activated significantly more for the P/I than for the temporal task (medial BA 10: *t* = −3.34, d.f. = 15, *p* = 0.004; medial ventro-caudal PFC: *t* = −2.08, d.f. = 15, *p* = 0.05), whereas for items perceived at study they were activated less for the P/I task than for the temporal task (medial BA 10: *t* = 4.59, d.f. = 15, *p* < 0.001; medial ventro-caudal PFC: *t* = 3.16, d.f. = 15, *p* = 0.006).

##### 3.2.2.5 Direct comparison of the functional roles of lateral versus medial rostral PFC

In order to assess the evidence for functional segregation between lateral and medial subregions of BA 10, we directly compared activity associated with contrasts assumed to preferentially activate lateral regions with activity associated with contrasts assumed to preferentially activate medial regions, crossing region with contrast. In order to obtain unbiased estimates, signal was extracted from 5 mm-radius spheres centred on voxels identified from the previous study of Simons, Owen, et al. (2005) (left lateral: −30, 63, 0; right lateral: 33, 60, 12; medial: −9, 63, 21). We first compared source recollection in general to the effect of study condition, by extracting data associated with the following orthogonal contrasts: Any Source Task > New (associated with activity in lateral BA 10 in the present data) and Any Source Task on Items Imagined at Study > Any Source Task on Items Perceived at Study (associated with activity in medial BA 10 in the present data). In an ANOVA with factors Region (left lateral, medial) and Contrast, there was a significant Region × Contrast interaction (*F*_(1,15)_ = 5.79, *p* = 0.03). Results were similar using the data from right lateral BA 10 instead of left lateral BA 10 (*F*_(1,15)_ = 18.20, *p* = 0.001). Next we compared source recollection in general to the effect of source task by extracting data associated with the following contrasts: Any Source Task > New (associated with activity in lateral BA 10 in the present data) and P/I Source > Temporal Source (assumed to preferentially involve medial BA 10, e.g. Simons et al., 2006). Again there was a significant Region × Contrast interaction for both left lateral compared to medial BA 10 (*F*_(1,15)_ = 19.78, *p* < 0.001), and for right lateral compared to medial BA 10 (*F*_(1,15)_ = 33.39, *p* < 0.001). These results establish distinct functional roles of lateral and medial BA 10 (guarding against the possibility of different contrasts activating different regions due to thresholding artifacts), with medial regions of BA 10 modulated by both study condition (greater for imagined than perceived items) and source task (greater for P/I rather than temporal source recollection).

##### 3.2.2.6 “Imagined-to-perceived” misattribution errors

**Fig. 4:**
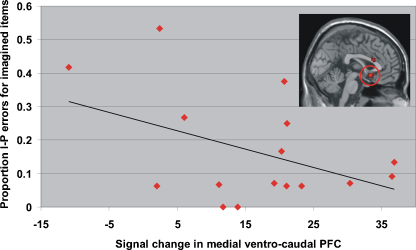
Scatter plot illustrating the correlation across participants between reduced activation in medial ventro-caudal PFC and likelihood of misattributing stimuli that were imagined at study and test as having been perceived.

Simons et al. (2006) reported that activation in medial rostral PFC was significantly negatively correlated with the likelihood of misattributing imagined items as having been perceived. Therefore we undertook correlation analyses exploring the relationship between the proportion of imagined items endorsed as perceived across subjects (*M* = 0.13, S.D. = 0.10) and the two regions of medial rostral PFC activated in our study. These regions were activated in two different contrasts, therefore signal change in each region was extracted from a 5 mm-radius sphere centred on the peak coordinate in the contrast which initially yielded the activation. There was no correlation between signal change and this type of attribution error in either right medial BA 9/10 (*r* = −0.21, n.s.) or left medial BA 10 (*r* = 0.08, n.s.). However there was a relationship between this kind of error and activation in the medial ventro-caudal (BA 25) region activated in the interaction between source task and study condition (see Fig. 4 and Table 5). Whilst there was no correlation with the proportion of imagined items endorsed as perceived overall (*r* = −0.2, n.s.), when analysis was restricted to those items also imagined at test (*M* = 0.16, S.D. = 0.16) this correlation approached significance (Fig. 3; *r* = −0.44, *p* = 0.08). Our results indicated that the lower the signal in this region, the more likely our subjects were to make this type of “imagined-to-perceived” misattribution error.

## 4 Discussion

This experiment used event related fMRI to contrast two source tasks hypothesised to recruit regions of rostral PFC. One required recollection of whether target items had previously been perceived or imagined by the participant (a reality monitoring task), and the other required recollection of which of two temporally distinct lists target items had been presented in (a temporal source monitoring task). The results revealed regions of bilateral rostrolateral PFC that were involved in both types of source recollection. The pattern of activation for *medial* rostral PFC however was quite different: subregions of medial rostral PFC were preferentially activated when participants were recollecting source information about items they had imagined at study. Specifically, a region of right medial rostral PFC was activated when participants retrieved either type of source information (P/I or temporal) relating to items imagined at study, and a region of left medial rostral PFC was preferentially activated when participants were required to determine the perceived/imagined status of items imagined at study. Direct comparison of the patterns of medial and lateral rostral PFC activation revealed region × condition interactions in which lateral rostral activation was greatest in contrasts emphasising general source recollection, whereas medial rostral activation was highest in contrasts emphasising source recollection associated with self-generated information, i.e. in contrasts isolating either P/I (rather than temporal) source recollection, or source recollection associated with items imagined at study (rather than items perceived at study). Consistent with previous studies in this area, activations in anterior PFC were not attributable to task difficulty, as estimated by accuracy and reaction time (Dobbins et al., 2002; Dobbins & Wagner, 2005; Simons, Owen, et al., 2005; Simons, Gilbert, et al., 2005; Simons et al., 2006, 2008). Analysis also revealed a region of medial ventro-caudal PFC, in which signal change was associated with the likelihood of misattributing imagined items as having been perceived in the external world. We will discuss first the lateral rostral findings, then the medial rostral findings, and finally the role of medial ventro-caudal regions in distinguishing perceived from imagined stimuli.

### 4.1 Lateral rostral PFC: a non-specific role in recollection of source information?

The present results suggest that regions of rostrolateral PFC are involved in recollection of several types of source information. Regions of left and right lateral rostral PFC were activated in retrieval of P/I source and in retrieval of temporal source information. This general pattern of lateral rostral PFC activation replicates previous findings associating lateral BA 10 with a range of source monitoring tasks including recollection of spatial context (Mitchell et al., 2004; Simons, Owen, et al., 2005; Simons et al., 2006), temporal context (Simons, Gilbert, et al., 2005), task context (Dobbins et al., 2002; Dobbins & Wagner, 2005; Kahn, Davachi, & Wagner, 2004; Rugg et al., 1999; Simons, Owen, et al., 2005; Simons, Gilbert, et al., 2005) size context (Dobbins & Wagner, 2005; Ranganath et al., 2000), voice and colour context (Fan et al., 2003), word/picture context (Mitchell et al., 2004; Nolde et al., 1998), perceived/imagined reality monitoring (Simons et al., 2006, 2008) and self/other reality monitoring (Simons et al., 2008). Our left lateralised activations were also very similar to those suggested by Dobbins and Wagner (2005) to be part of a domain-general control network recruited during any kind of contextual remembering. These regions appear to support processes related to recollection of many types of source information, and there is some evidence to suggest that they are involved in the early stages of retrieval, such as retrieval orientation or cue specification (Simons, Gilbert, et al., 2005; Simons et al., 2008). The suggestion that bilateral rostral PFC, along with dorsolateral and ventrolateral regions of PFC, is involved in recollection of source information in general is supported by evidence that patients with frontal lobe lesions have difficulties in a range of source monitoring tasks (e.g. Janowsky, Shimamura, & Squire, 1989; Kopelman, Stanhope, & Kingsley, 1997; Milner, Petrides, & Smith, 1985; Shimamura, Janowsky, & Squire, 1990).

Moreover the involvement of lateral rostral PFC in source memory tasks is consistent with accounts of the information processing role of lateral rostral PFC that have emerged from outside the source memory literature. For instance, both Burgess and colleagues and Christoff and colleagues have proposed a role for lateral rostral PFC in the attending requirements made by various forms of “stimulus-independent” thought (i.e. self-generated and maintained cognition: Burgess, Dumontheil, & Gilbert, 2007; Burgess, Gilbert, & Dumontheil, 2007; Christoff & Gabrieli, 2000; Christoff, Ream, Geddes, & Gabrieli, 2003; Gilbert, Frith, & Burgess, 2005; Gilbert, Spengler, Simons, Frith, & Burgess, 2006). Indeed Burgess, Dumontheil, et al. (2007) and Burgess, Gilbert, et al. (2007) have argued on these grounds that source memory tasks should provoke engagement of lateral rostral PFC (because they require the orientation of attention towards internally maintained representations). In addition, several authors have proposed that lateral rostral PFC plays a critical role in the co-ordination and management of subgoals, and integrating recovered information with a final goal (Badre & Wagner, 2004; Braver & Bongiolatti, 2002; Koechlin, Basso, Pietrini, Panzer, & Grafman, 1999; Ramnani & Owen, 2004). This account fits well with the involvement of rostral PFC in source recollection tasks, which presumably require integration of recovered source information with item information.

### 4.2 Medial rostral PFC: a role in recollection of source related to self-generated stimuli?

This study also tested the theory that some regions of rostral PFC may be differentially engaged by the requirement to recollect different kinds of contextual detail, and specifically that medial rostral PFC might be associated with recollection of whether items had previously been imagined or perceived (reality monitoring). For example, Simons et al. (2006) directly compared perceived/imagined (P/I) source recollection to recollection of which side of the screen stimuli had been presented on (spatial context), and reported that medial rostral PFC was more activated by the P/I task than by the spatial context task. The design of the present experiment also allowed us to separate activation associated with source recollection for items that had been imagined at study and items that had been perceived at study. Our results indicated that medial rostral PFC regions may in fact be modulated not only by the type of source task that is being conducted, but also by the type of memory product one is working with (previously imagined or previously perceived). We found that medial rostral PFC was preferentially activated when participants were reflecting on stimuli they had previously imagined, suggesting that these regions may be critically sensitive to recollection of previously imagined, or self-generated, material.

A region of right medial BA 9/10 was activated when participants completed either source task (P/I or temporal) on a previously imagined stimulus, and a region of left medial BA 10 was preferentially activated when participants were required to determine the perceived or imagined status of a previously imagined word. These regions were rather more superior, but otherwise similar to medial rostral PFC regions previously associated with recollection of “task context” (i.e. what type of judgement the participant had previously carried out on the stimuli: Dobbins & Wagner, 2005; Simons, Owen, et al., 2005; Simons, Gilbert, et al., 2005), recollection of source for self-generated rather than experimenter-generated words (Vinogradov et al., 2006), and recollection of whether stimuli had been perceived or imagined (Simons et al., 2006). It is tempting to speculate that there may be a common processing requirement presented by these various tasks. An obvious candidate might be that they all require, to some extent, retrieval of self-generated rather than externally derived material. This fits well with the source monitoring framework (Johnson et al., 1993; Johnson, 1997), which states that we are able to attribute memories to a perceived or an imagined source on the basis of the type of information that they are associated with. Perceived items will be associated with perceptual information (e.g. colour or sound), and contextual (spatial and temporal) information, whereas imagined events will be associated with cognitive operations (e.g. the reflective and elaborative processes that took place when the memory was established). It appears to be the recollection of cognitive operations which results in activations in medial rostral PFC.^1^

Indeed, it is possible that the two medial rostral PFC activations identified here may reflect subtly different types of processing. There is considerable evidence that BOLD signal changes can be provoked in caudal medial aspects of rostral PFC by “mentalising” tasks—the reflection on one's own emotions and mental states, or those of others (Frith & Frith, 2003; Gilbert, Spengler, Simons, Steele, et al., 2006; Gilbert et al., 2007; Gusnard & Raichle, 2001). Our right medial activation, with a *y* coordinate of 48, was very close to this mentalising region. It was also very close to one of the regions reported by Vinogradov et al. (2006) as being associated with correct recollection of source for self-generated rather than experimenter-generated words. They attributed their results to the greater self-referential processing and sense of cognitive agency associated with these items. Simons et al. (2008) have suggested a similar account: that more caudal regions of rostral PFC may be particularly involved in self/other distinctions, in comparison to the rostral loci associated with perceived/imagined distinctions. On these grounds one might speculate that our more caudal right medial activation may reflect processes related to self-reflection or similar metacognitive processes, whereas our more anterior left medial activation may reflect processing specifically involved in identifying previously imagined events as imagined.

### 4.3 Medial ventro-caudal PFC and recollection of perceived/imagined source information

Analysis also revealed a region of medial ventro-caudal PFC (BA 25) in which signal change was negatively correlated with the proportion of imagined items endorsed as perceived across subjects. Damage to medial ventro-caudal PFC and nearby basal forebrain structures is known to be implicated in confabulation, the unknowing production of erroneous memories and occasionally bizarre false accounts (Damasio, Graff-Radford, Eslinger, Damasio, & Kassell, 1985; DeLuca & Diamond, 1995; Fischer, Alexander, D’Esposito, & Otto, 1995; Gilboa et al., 2006; Irle, Wowra, Kunert, Hampl, & Kunze, 1992; Turner, Cipolotti, Yousry, & Shallice, 2008). Moreover, confabulating patients are known to have difficulty with reality monitoring tasks (Dalla Barba, Capelletti, Signorini, & Denes, 1997) and sometimes show a bias towards misidentifying imagined events as real (Johnson, O’Connor, & Cantor, 1997; Turner et al., in preparation). Therefore the pattern revealed by our correlation is what one would expect if confabulation is associated with reduced activity in medial ventro-caudal PFC. Our results appear to provide converging evidence from neuroimaging implicating this region in the support of processing involved when we distinguish imagined from real events. The mechanistic role of this region in confabulation and misattribution errors is at present unclear. However one possibility is that this region is particularly crucial for a type of monitoring in which imagined items are identified as less “real” or reliable than other information. This may be secondary to a “feeling of rightness” monitoring process which has been attributed to ventromedial regions by Gilboa et al. (2006). Alternatively it may be involved in a process whereby imagined or perceived source is established via a matching of events to our internal predictions. Whereas real events are assumed always to engender a slight mismatch or prediction error, leading to arousal, imagined items will lack this response, enabling them to be identified as internal (Frith, 2007).

In summary, this study confirms previous reports implicating rostral PFC in source recollection tasks, but also indicates important functional differences between lateral and medial regions. Our results, in combination with previous studies, suggest three conclusions. First, that lateral regions of rostral PFC are involved in several different types of source recollection. We have drawn from accounts outside the source memory literature to speculate that this may reflect the attentional requirements such tasks make for orienting towards internally maintained representations. Second, we found that medial regions of rostral PFC were preferentially activated when participants were required to recollect source details concerning self-generated information. We have drawn parallels between these BOLD signal changes and the patterns of activation seen during tasks that require “mentalising” or other metacognitive or self-referential processing. Third, we have shown that medial ventro-caudal/basal forebrain regions are recruited by healthy participants when determining whether imagined stimuli have been externally perceived or only imagined, and that reduced activity in this region is associated with making confabulatory errors.

## Figures and Tables

**Table 1:** Accuracy and reaction time data

	Accuracy		Reaction time (ms)	
	Mean	Standard error	Mean	Standard error
Study phase				
Perceived	0.98	0.01	1818	161
Imagined	0.92	0.01	2505	106

Test phase				
Effect of study condition				
Perceived	0.81	0.02	3178	117
Imagined	0.82	0.02	3164	104

Effect of test condition				
Perceived	0.82	0.02	3035	112
Imagined	0.81	0.02	3307	99

Effect of task				
Perceived/imagined source	0.85	0.02	3314	115
Temporal source	0.77	0.03	3028	99

**Table 2:** Common regions activated in correct source recollection trials **(**perceived/imagined and temporal source) vs. correct rejection of new items

Brain region	Coordinates			*Z*	Voxels
	*x*	*y*	*z*		
Left lateral rostral PFC (BA 10)	−30	60	6	3.73	14
Right lateral rostral PFC (BA 10)	27	54	3	4.11	14
Left dorsolateral PFC (BA 46)	−42	21	27	5.13	101
Left insula/inferior PFC (BA 13/47)	−27	27	−3	4.15	13
Right insula/inferior PFC (BA 13/47)	30	27	3	4.66	13
Left superior medial PFC (BA 8)	−6	21	51	3.97	27
Left lateral premotor cortex (BA 6)	−27	12	60	4.47	86
Left lateral inferior parietal cortex (BA 40)	−39	−54	45	5.24	530
Right lateral inferior parietal cortex (BA 40)	36	−51	45	4.81	167
Left medial occipito-parietal cortex (BA 7/19)	−9	−69	30	5.82	577
Left medial occipital cortex (BA 18)	−15	−84	−6	4.73	104
Left caudate	−9	9	0	3.77	35
Cerebellum	12	−81	−27	3.89	13

**Table 3:** Regions exhibiting significantly greater activation for correct recollection of temporal source than perceived/imagined source

Brain region	Coordinates			*Z*	Voxels
	*x*	*y*	*z*		
Left lateral rostral PFC (BA 10)	−45	51	0	4.19	86
Right lateral rostral/orbital PFC (BA 10/11)	33	45	−6	4.31	10
Right frontal cortex	27	−3	36	3.75	17
Right lateral parietal cortex (BA 7)	45	−72	45	3.86	11
Left caudate	−9	15	−3	4.08	11
Left putamen	−30	−6	−3	4.63	131
Right putamen	27	0	−6	5.81	158
Cerebellum	−21	−72	−45	5.21	25
Cerebellum	21	−66	−45	4.46	10
Cerebellum	15	−66	−18	3.87	42

**Table 4:** Regions exhibiting significantly greater activation for source recollection associated with items imagined at study than items perceived at study

Brain region	Coordinates			*Z*	Voxels
	*x*	*y*	*z*		
Left lateral rostral PFC (BA 10)	−39	45	24	3.48	10
Right medial PFC (BA 9/10)	12	48	27	3.76	13
Left dorsolateral PFC (BA 9)	−33	27	42	4.28	33
Right ventrolateral PFC (BA 44)	45	15	12	4.31	15
Right lateral PFC (BA 8/9)	57	12	42	3.76	12
Right lateral PFC (BA 8)	33	39	45	4.53	21
Left lateral premotor cortex (BA 6)	−30	−6	57	3.97	41
Left medial premotor cortex (BA 6)	−9	−18	−51	3.62	18
Right lateral premotor cortex (BA 6)	36	−9	36	3.82	13
Left lateral temporal cortex (BA 22/42)	−51	−36	9	4.12	15
Left lateral temporal cortex (BA 21)	−63	−45	−6	3.66	22
Right lateral temporal cortex (BA 37)	57	−57	−12	3.63	12
Right lateral temporal cortex (BA 42)	60	−27	15	4.01	16
Left lateral inferior parietal cortex (BA 40)	−39	−36	54	4.14	44
Left lateral inferior parietal cortex (BA 40)	−63	−33	24	3.63	10
Left lateral inferior parietal cortex (BA 40)	−42	−63	42	4.50	51
Left posterior cingulate cortex (BA 31)	−3	−54	27	4.10	48
Right lateral occipital cortex (BA 19)	42	−81	33	4.24	10
Right medial occipital cortex (BA 19)	6	−84	42	3.62	38
Cerebellum	18	−69	−30	4.47	12
Cerebellum	−15	−57	−27	3.56	11

**Table 5:** Regions of significant activation in the interaction [Imagined at study (P/I–temporal source)] − [Perceived at study (P/I–temporal source)] indicating activation specifically associated with identifying whether previously imagined words had been seen or imagined

Brain region	Coordinates			*Z*	Voxels
	*x*	*y*	*z*		
Left lateral rostral PFC (BA 10)	−30	45	30	3.68	15
Left medial rostral PFC (BA 10)	−12	57	30	4.53	32
Medial ventro-caudal PFC/basal forebrain (BA 25)	0	15	−9	4.09	14
Left anterior cingulate cortex (BA 24)	−6	24	18	3.42	11
Left dorsolateral PFC (BA 9)	−21	39	39	3.45	11
Right medial PFC (BA 8)	6	45	45	4.20	42
Right lateral temporal cortex (BA 22)	60	−9	6	3.46	12

## Footnotes

1 Given this hypothesis it is intriguing that source recollection for items perceived at study in the current paradigm was not associated with posterior activation, reflecting retrieval of perceptual information (e.g. Kensinger & Schacter, 2006). In fact the perceived > imagined contrast yielded no activations, whilst the imagined > perceived contrast was associated with several large posterior activations. Imagined items were associated with longer RTs at study than perceived items, and this greater posterior activation at test might therefore be related to the fact that subjects had previously spent more time looking at these stimuli. However further studies targeting these processes will clearly be required in order to explain this pattern of data.
